# Protein transduction domain-mediated influenza NP subunit vaccine generates a potent immune response and protection against influenza virus in mice

**DOI:** 10.1080/22221751.2020.1812436

**Published:** 2020-09-02

**Authors:** Yuan Yin, BeiBei Li, Linting Zhou, Jian Luo, Xueying Liu, Shilei Wang, Qun Lu, Wensong Tan, Ze Chen

**Affiliations:** a Department of Clinical Laboratory, Shanghai TCM-Integrated Hospital, Shanghai University of Traditional Chinese Medicine, Shanghai, People’s Republic of China; b Shanghai Institute of Biological Products, Shanghai, People’s Republic of China; c State Key Laboratory of Bioreactor Engineering, East China University of Science and Technology, Shanghai, People’s Republic of China; d College of Life Science, Hunan Normal University, Changsha, People’s Republic of China

**Keywords:** Influenza virus, universal vaccine, nucleoprotein, protein transduction domain, nucleoprotein, cross-protection

## Abstract

The nucleoprotein (NP) is a highly conserved internal protein of the influenza virus, a major target for universal influenza vaccine. Our previous studies have proven NP-based subunit vaccine can provide partial protection in mice. It is reported that the protein transduction domain (PTD) TAT protein from human immunodeficiency virus-1 (HIV-1) is able to penetrate cells when added exogenous protein and could effectively enhance the immune response induced by the exogenous protein. In present study, the recombinant protein TAT-NP, a fusion of TAT and NP was effectively expressed in *Escherichia coli* and purified as a candidate component for an influenza vaccine. We evaluated the immunogenicity and protective efficacy of recombinant influenza TAT-NP vaccine by intranasal immunization. In vitro experiments showed that TAT-NP could efficiently penetrate into cells. Animal results showed that mice vaccinated with TAT-NP could not only induce higher levels of IgG and mucosal IgA, but also elicit a robust cellular immune response. Moreover, the TAT-NP fusion protein could significantly increase the protection of mice against lethal doses of homologous influenza virus PR8 and could also provide mice protection against a lethal dose challenge against heterosubtypic H9N2 and H3N2 influenza virus. In conclusion, the recombinant TAT-NP might be a universal vaccine candidate against influenza virus.

## Introduction

Influenza virus is one of the most common causes of serious respiratory diseases and represents an important economic burden worldwide [1]. Currently, vaccination remains the most effective strategy to prevent disease caused by influenza. However, seasonal influenza vaccines could only induce narrow-spectrum and strain-specific immune responses. The vaccine components should be reformulated each year due to antigenic drift of influenza virus strains. Mismatches between the vaccines and circulating strains often lead to high morbidity. During the 2014–2015 influenza season, due to the antigenic drift of H3N2, seasonal influenza vaccine effectiveness against antigenically drifted A(H3N2) strains was significantly reduced [2]. In addition, different subtypes of influenza viruses are susceptible to genetic reassortment after infecting the same host, and the new influenza strains generated may also cause unpredictable influenza epidemics and even pandemics. In recent years, cross-species transmission of the influenza virus has led to the spread of many avian influenza viruses into the human population. At present, many avian influenza viruses capable of infecting humans have been found, such as H5N1, H5N6, H6N1, H7N2, H7N3, H7N7, H7N9, H9N2, H10N7 and H10N8 [3]. These emerging and re-emerging influenza viruses have posed a great threat to human health [4,5]. Therefore, development of a universal influenza vaccine capable of inducing cross-protection among different subtypes of influenza viruses has become an important trend in rapidly responding to the epidemics of new influenza viruses.

Currently, the conserved proteins of influenza virus, such as stem region of hemagglutinin (HA), nucleoprotein (NP) and matrix protein (M), are the main targets to develop universal influenza vaccines [6,7]. Influenza virus nucleoprotein, type-specific, is highly conserved in the influenza virus. NP is conserved among different viral strains by more than 90% [8,9]. After influenza virus infection, NP is a major antigen recognized by cytotoxic T lymphocytes (CTL) [10]. NP-specific CTL can promote lysis of the virus-infected cells by recognizing the NP antigenic peptides presented by MHC-I molecules on the surface of virus-infected cells. Thus, they contribute to the clearance of the virus and prevent the spread of viral infection [11]. In addition, NP-specific CD4^+^ T cell responses and antibody responses also play a certain role in cross-protection [12]. Therefore, induction of strong NP-specific immune responses, especially cellular immune responses, becomes one of the targets for design of NP-based broad-spectrum influenza vaccines.

In 1988, Green and Frankel et al. reported for the first time that the TAT protein of HIV-1 was capable of transmembrane penetration into the cells, and subsequent studies showed that HIV-TAT can also mediate the entry of many exogenous biological macromolecules into cells [13,14]. This peptide with a protein transduction effect is called protein transduction domain (PTD). Currently, the identified short peptides with protein transduction effect include HSV-1 VP22, MAP and PEP-1 [15]. Further studies show that TAT plays a role in protein transduction, mainly for its 47–57 amino acids. It is reported that TAT protein is able to enter cells when combined with exogenous antigens and induce enhanced MHC I restrictive CD8^+^ CTL response [16–18].

In this study, we selected the conserved internal protein NP of the influenza virus, with fusion expression of TAT at the N-terminal of NP to express the recombinant TAT-NP protein in *Escherichia coli*. We aimed to evaluate the immune protection of recombinant protein vaccines. And the results showed that TAT-NP could provide enhanced protection against lethal dose challenge with homologous and heterosubtypic influenza virus in mice.

## Materials and methods

### Preparation of rTAT-NP

The plasmid pET28a-NP encoding the NP gene derived from influenza virus A/PR8/34(H1N1) was constructed in our previous study [19]. The segment TAT (YGRKKRRQRRR) was ligated to the N-terminal of NP by overlap extension PCR. The TAT-NP gene was inserted into expression vector to construct the recombinant vector pET28a-TAT-NP. The recombinant plasmid was transformed into *E. coli* BL21(DE3) strain for expression. The target protein was purified using AKTA Purifier (GE) with a Ni-chromatography column (GE). The purified proteins were assessed by SDS-PAGE and Western blotting. Protein concentration was measured using Bradford reagent (Thermo) and stored at −70°C. The production of recombinant protein was 2–5 mg/L.

### Cellular uptake of TAT-NP

293 T cells were incubated with either TAT-NP or NP (10 or 20 μg/mL) at 37°C for 2 h. The cells were then washed three times with PBS and fixed with 4% Paraformaldehyde for 30 min. After washing three times with PBST (0.1% Tween-20), and blocking with PBST containing 5% BSA for 30 min for cell permeabilization, anti-His antibody was added. After incubation at 37°C, the cells were washed three times with PBST. FITC conjugated goat anti-mouse IgG was added. After incubation 37°C in dark, the cells were washed with PBS and viewed with fluorescence microscope.

### Viruses, mice and cells

Influenza virus included mouse-adapted A/PR8/34(H1N1), A/Chicken/Jiangsu/7/2002 (H9N2) and A/Guizhou/54/1989 (H3N2) viruses were used in this study as described in our previous studies [20]. Live-virus experiments were performed in Biosafety Level 2 facilities under governmental and institutional guidelines in SIBP (Shanghai Institute of Biological Products). Madin-Darby canine kidney (MDCK; ATCC CCL-34) cells were purchased from the American Type Culture Collection (ATCC) and were grown Dulbecco’s modified Eagle’s medium (DMEM; Gibco) supplemented with 10% fetal bovine serum (FBS; Gibco) and 100 units/mL of penicillin and 100 μg/mL streptomycin (Pen-Strep; Gibco).

Groups of female BALB/c (H-2d) mice (specific-pathogen-free, SPF) of 6–8 weeks old were purchased from Shanghai Laboratory Animal Center, China. All mice were housed in the Animal Resource Center at SIBP and maintained in SPF conditions. Animals were anesthetized by intraperitoneal injection of 1% Pelltobarbitalum Natricum (60 mg/kg bodyweight). All experiments involving animals have been approved by Animal Care Committee of SIBP. Mice that lost over 30% of their initial body weight were scored dead and humanely euthanized.

### Immunization and challenge

For a homologous protection study, BABL/c mice (*n *= 19/group, female, 6–8 weeks old) were randomly divided to 6 groups. Mice were anesthetized, followed by immunized intranasally 3 times with 2 weeks interval containing different doses of 40 μL NP or TAT-NP. The control group was received PBS. Two weeks after the last immunization, animals were anesthetized and intranasally challenged with 20 μL of the viral suspension containing 10 × LD_50_ of A/Puerto Rico/8/34 (H1N1). After viral challenge (3 days), Lung tissues were collected from 3 mice/group for detecting virus titers and 3 mice/group for pathological investigation. Mice (*n *= 10/group) were monitored daily to record clinical symptoms, weight loss and mortality. For a heterosubtypic protection study, a total of 78 mice were randomly divided into six groups (*n* = 13/group). Mice were intranasally immunized with either NP or TAT-NP 3 times with 2 weeks interval. The control group was received PBS. Two weeks after the last immunization, the mice were intranasally inoculated with 10 × LD_50_ of A/Chicken/Jiangsu/7/2002 (H9N2) or 10 × LD_50_ A/Guizhou/54/1989 (H3N2) respectively. The mice were observed for 14 days after viral challenge, during which weight loss and survival rate were recorded. Virus titers in the lungs collected at day 3 post-challenge were tested via TCID_50_ assay. All animal experiments were repeated three times in mice.

### Specimen collection

Blood samples (100 μL) of three mice in each group were collected on day 14, 28 and 42 via retrobulbar bleeding and used for NP-specific IgG, IgG1 and IgG2a Ab assays, respectively. Two weeks after the last immunization, spleens were isolated from three mice in each group and single cell suspensions were prepared as described and monitored for the presence of antigen-specific T cells [19]. A syringe needle with 1 mL of PBS was inserted three times into the nasopharynx to collect the nasal lavage fluid (NAL). The NAL was centrifuged to remove cellular debris and used for IgA Ab assays. Three mice from each group were anesthetized and killed by cervical dislocation three days after challenge. The lungs were removed aseptically and injected with a total of 2 mL of PBS containing 0.1% BSA. Bronchoalveolar lavage fluid (BALF) was collected and stored at −70°C until processing for virus titration.

### Enzyme-linked immunosorbent assay (ELISA)

The NP-specific antibodies in the serum (IgG, IgG1, IgG2a) and NAL (IgA) were determined by ELISA as described in our previous studies [19,20]. ELISA plates (Corning) were coated overnight at 4°C with purified NP (5 μg/mL). Serum and NAL was diluted according to the results of pre experiment, and then serially diluted in 1:2 steps. Goat anti-mouse IgG Ab (γ-chain specific) (KPL), goat anti-mouse IgG1 Ab (KPL), goat anti-mouse IgG2a Ab (KPL), and goat anti-mouse IgA (α-chain specific) (KPL) conjugated with horseradish peroxidase (HRP) was used as the secondary antibody. The signal was developed using tetramethylbenzidine (TMB) as the substrate. The optical density was read at 450 nm. End-point ELISA titers were expressed as the reciprocal of the highest sample dilution that yielded an OD ≥ mean+2 × SD of control mouse serum.

### Gamma interferon (IFN-γ) ELISpot assay

The vaccine specific cellular immune response in mice was determined using ELISpot kit (Mabtech AB) as described in our previous study [19,20]. Spleen cells were isolated two weeks after the last immunization. Pooled spleen cells (5 × 10^5^ cells/well) were stimulated either with MHC-I peptide and a pool of three MHC-II peptide from influenza virus NP as previously described in our study [21]. The numbers of colour spots representing IFN-γ-secreting T cells were counted using an ELISpot reader (Bioreader 4000; Bio-Sys, Germany). The number of peptide-reactive cells was represented as spot-forming cells (SFCs) per 10^6^ splenocytes and was calculated by subtracting spot numbers in medium only wells from spot numbers in peptide containing wells.

### Viral titration

Three challenged mice were euthanized at day 3 post-challenge, lungs were isolated and the BALF was prepared as above. Virus titers were determined in triplicate by titration of the BALF on MDCK cells. Briefly, monolayers of cells were infected for 1 h with 100 μL of serial 1:10 dilutions of the bronchoalveolar wash in a 96-well plate in serum-free DMEM medium containing penicillin and streptomycin (Gibco). Following infection, the medium was replaced by medium containing 2 mg/mL of TPCK-treated trypsin (Sigma–Aldrich). Endpoint virus titers were determined after three days, as described by Reed and Muench. The virus titer was expressed as 50% tissue culture infection dose (TCID_50_) and represented by the mean ± SD of virus titer per mL of specimens from three mice in the group.

### Hematoxylin and eosin staining

Three days post-challenge, lungs of three infected mice were isolated, immediately fixed in 10% neutral-buffered formalin, and embedded in paraffin. Sections (4∼6 μm) were mounted on slides, stained with H&E (Shanghai Biyuntian Biological Co., Ltd.), and observed by microscopy. Three random sections of each lung sample were examined. Three visual fields were evaluated in each pathological section. Histopathological changes were examined by a pathological investigator, who was unaware of the samples’ origin.

### Statistics

Comparison of antibody titers, lung virus titers and cytokine response between two groups was evaluated with the two-tailed Student’s *t*-test. Survival rates were plotted as Kaplan-Meier curves and analyzed with the log-rank test Statistical analysis. The data was were performed with GraphPad Prism version 8.0. *P-*values of less than 0.05 (*P* < 0.05) were considered to be statistically significant. Results are expressed as the means ± SD.

## Results

### Preparation and characterization of recombinant TAT-NP

The recombinant fragment TAT-NP was inserted into pET28a with His-Tag, and expressed in *E. coli* BL21(DE3) strain. The recombinant protein was purified by His-Tag chromatography and confirmed by western-blot (Figure S1).

### Ability of TAT-NP fusion protein to penetrate cells

To evaluate whether TAT could enhance the penetration of exogenous protein into cells, we incubated 10 or 20 μg/mL of NP protein and TAT-NP protein with 293 T cells for 2 h respectively, and evaluated the effect of TAT protein transduction using indirect immunofluorescence assay, with PBS as the control. As shown in Figure S2, strong green fluorescence was widely distributed in the 293 T cells incubated with 10 or 20 μg/mL TAT-NP protein after the same incubation time, and only weak fluorescence was observed in the cells incubated with the same dose of NP protein. The result indicates that TAT could effectively transduce the fused protein into the cells.

### Mice vaccinated with TAT-NP were completely protected against challenge with a homologous influenza virus

**Figure 1.**
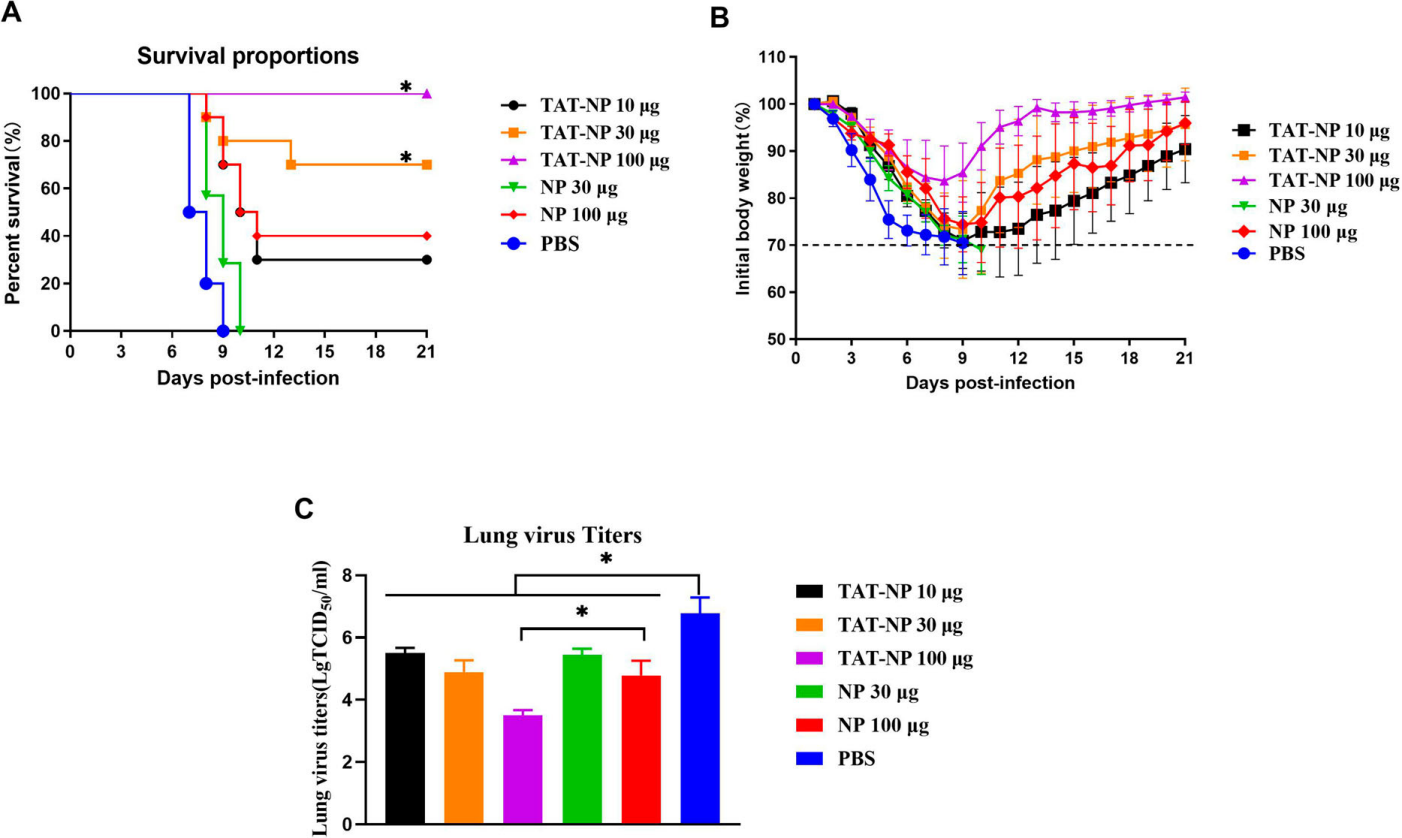
Protection of vaccinated mice against challenge with homologous virus. BABL/c mice (*n *= 114, female, 6–8 weeks old) randomly were divided to six groups. Mice were anesthetized, followed by immunized intranasally 3 times with 2 weeks interval containing different doses of 40 μL NP or TAT-NP. The control group was received PBS. Two weeks after the last immunization, mice were anesthetized and intranasally challenged with 20 μL of the viral suspension containing 10 × LD_50_ of A/Puerto Rico/8/34 (H1N1). Survival rates (A), body weight changes (B) of ten mice were monitored daily for 3 weeks after challenge. Weight loss is shown mean of the group with error bars representing SD (*n *= 10). The survival rates were evaluated by log-rank (Mantel–Cox) test. * indicates significant differences compared with the same dose NP group (*P *< 0.05). The lung virus titers (C) are expressed as mean ± SD of three tested mice in each group. The *t*-test was used to determine difference in viral load. * indicates significant differences (*P *< 0.05).

To determine the protective efficacy of TAT-NP vaccination, mice were vaccinated with 10, 30 and 100 μg of TAT-NP protein respectively, with 30 and 100 μg of NP protein and PBS as the control groups for vaccination three times at two-week intervals. Mice were challenged with homologous 10 × LD_50_ A/PR/8/34 (H1N1) two weeks after the last vaccination. The trachea and lungs were randomly taken from 3 mice in each group three days after challenge to determine the virus load in the BALF. On the second day after the challenge, the mice in all groups showed such clinical symptoms as pilomotor fur, anorexia and weight loss. As shown in Figure 1(A), mice vaccinated three times with 100 μg NP alone could not be completely protected, with the survival rate of only 40% (4/10), while mice vaccinated with 100 μg TAT-NP could be completely protected, with the survival rate of 100% (10/10). Meanwhile, 30 μg TAT-NP showed a better protection than the same dose NP group (7/10 vs. 0/10).

Mice in all groups had different degrees of weight loss. When weight loss over 30% of their initial weight, the mice were euthanized. The same dose TAT-NP group showed less weight loss than the NP group, and the PBS control group showed maximum weight loss. The maximum weight loss of TAT-NP 100 μg group was only 15.6%, and the maximum weight loss of NP 100 μg group was up to 25.5%. TAT-NP group could start weight recovery more rapidly. In addition, the lung virus titer of mice was also detected 72 h after infection. As shown in Table 1 and Figure 1(C), virus titer in the lungs of mice in the NP and TAT-NP groups was significantly lower than that in the control group (*P* < 0.05), and the lung virus titer in the 100 μg TAT-NP group was significantly lower than that in the same dose NP group (*P* < 0.05). In addition, virus titer in the lungs of mice was well correlated with the protection provided by the vaccine to mice. Consistent with this result, the lung histopathology of mice vaccinated with TAT-NP was less severe than mice vaccinated with the same dose of NP and control group. Severe pathological damage of lung tissue was observed the mice, which included infiltration with inflammatory cells (Figure S3).

**Table 1.** Protection against lethal PR8 virus challenge in mice by administrated intranasally with NP or TAT-NP vaccine.

Group	Immunogen	Dose (μg)	Protection against challenge with PR8 virus	
			Lung virus titers (Log_10_TCID_50_/mL)	Survival mice/tested mice
A	TAT-NP	10	5.50 ± 0.17^b^	3/10^b^
B	TAT-NP	30	4.89 ± 0.38^b^	7/10^ab^
C	TAT-NP	100	3.50 ± 0.17^ab^	10/10^ab^
D	NP	30	5.44 ± 0.20^b^	0/10
E	NP	100	4.78 ± 0.48^b^	4/10^b^
F	PBS	−	6.78 ± 0.51	0/10

Note: BABL/c mice were randomly divided into six groups. Mice were immunized intranasally with NP or TAT-NP vaccine, whereas control animals received PBS. Two weeks after the last immunization, mice were challenged with a lethal dose (10 × LD_50_) of influenza PR8 virus. BALF of three mice per group was collected 3 days after the challenge. Virus titers in the lungs as determined by plaque assay. Results are expressed as mean ± SD of three tested mice in each group. Survival rate of mice (*n *= 10) was monitored for 21 days. The *t*-test was used to determine difference in viral load. The survival rates were evaluated by log-rank (Mantel–Cox) test.

^a^Displays statistically significant difference compared with the same dose NP group (*P* < 0.05).

^b^Displays statistically significant difference compared with the PBS group (*P* < 0.05).

In conclusion, the above results indicate that the candidate vaccine TAT-NP can provide mice with protection against lethal doses of a homologous influenza virus challenge without the use of an adjuvant.

### TAT-NP induces potent antigen-specific humoral immune responses at the systemic and mucosal level

**Figure 2.**
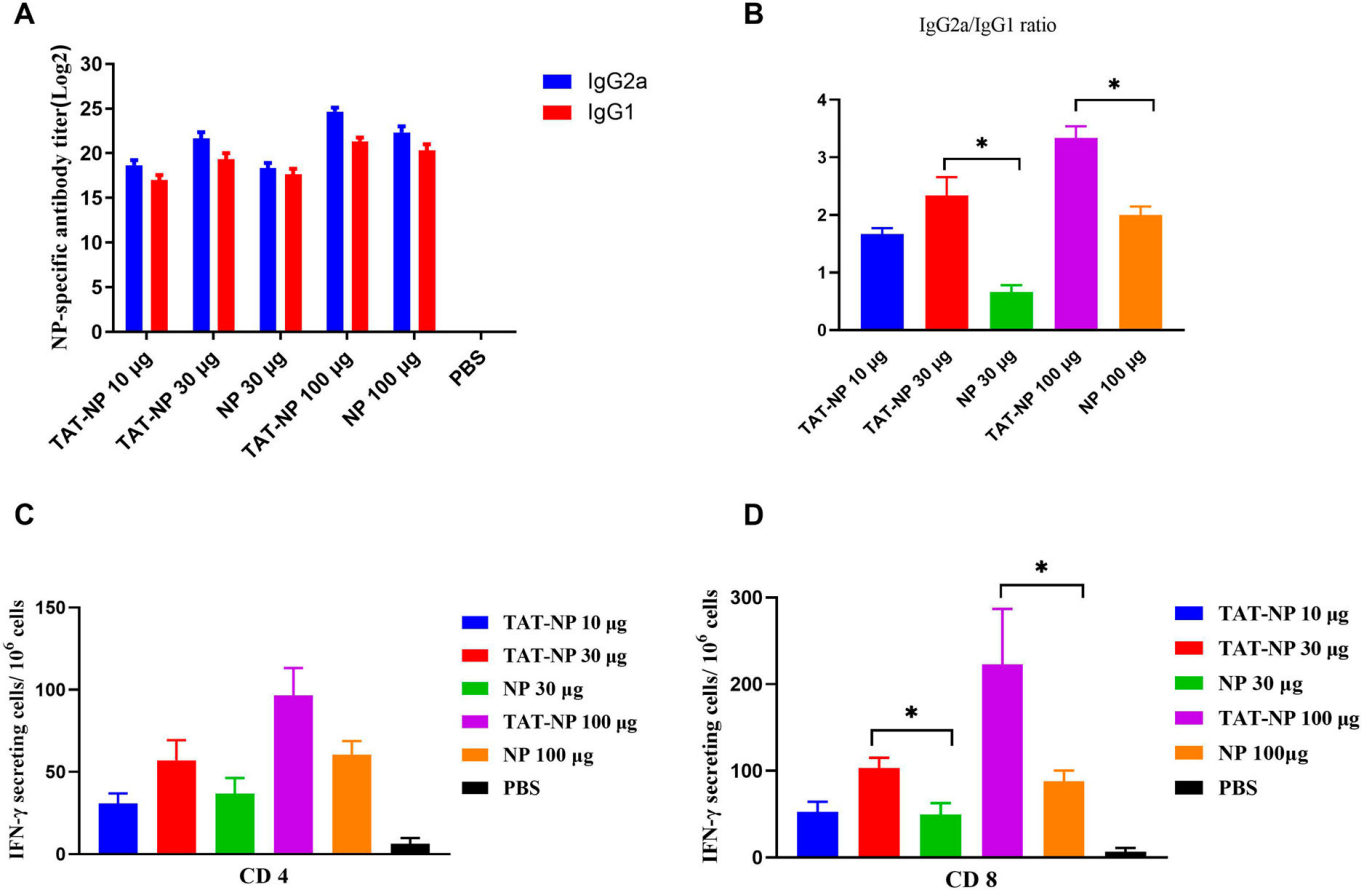
Humoral and cellular immune responses induced by TAT-NP vaccine. Anti-NP IgG was determined by ELISA on day 42. Mice were immunized three doses intranasally with NP or TAT-NP, whereas control animals received PBS, at an interval of 2 weeks. (A) The antibody responses of IgG1 and IgG2a detected in sera after the last immunization. End-point ELISA titers were expressed as the reciprocal of the highest sample dilution that yielded an OD ≥ mean+2 × SD of control mouse group. Results are expressed as mean ± SD of three tested mice in each group. (B) The ratio of IgG2a/IgG1 2 weeks after the last immunization. Two weeks after the last immunization, splenocytes were harvested and stimulated with peptides recognized by MHC class I and class II restricted epitopes of NP. Results are expressed as number of spots of cells producing cytokines per 10^6^ spleen cells. Detection of IFN-γ secreting CD4^+^ (C) and CD8^+^ (D) T cells. Data shown as mean numbers of spot-forming cells (SFCs) ± SD. *n* = 3 per group. Each sample was tested in triplicates. * indicates significant differences (*P *< 0.05, two-tailed Student’s *t*-test).

Humoral immune responses play an important role in clearing pathogen infections [22]. We assessed the systemic and mucosal immune response induced by TAT-NP in intranasally vaccinated mice. Six groups of BALB/c mice were vaccinated three times at two-week intervals. Two weeks after each vaccination, the anti-NP IgG titer was detected in serum by ELISA. As shown in Table 2, the anti-NP IgG titer increased with the number of vaccinations. After three vaccinations, NP-specific antibodies could reach high titers in all groups. The titer of anti-NP IgG of TAT-NP immunization was higher than in the NP group, but the differences were not significant. In addition, the antibody subtypes after the last vaccination was analyzed to detect the IgG1 and IgG2a titers in the serum. The results showed that high titers of IgG1 and IgG2a antibodies were induced in all groups (Figure 2(A)), and the ratio of IgG2a/IgG1 in TAT-NP group was higher than that in the NP group, indicating that more pronounced Th1-biased immune response was induced in the TAT-NP group (Figure 2(B)).

**Table 2.** Serum and mucosal antibody responses in mice.

Group	Immunogen	Dose (μg)	Ab response (ELISA, 2^n^)			
			Serum IgG (Day 14)	Serum IgG (Day 28)	Serum IgG (Day 42)	NAL IgA
A	TAT-NP	10	13.00 ± 1.00	18.33 ± 0.58	21.33 ± 0.58	5.33 ± 0.58
B	TAT-NP	30	14.30 ± 0.58^a^	19.33 ± 0.94	23.00 ± 0.00	6.00 ± 0.00^a^
C	TAT-NP	100	15.60 ± 1.52^a^	23.33 ± 0.67	25.67 ± 0.58	7.33 ± 0.58^a^
D	NP	30	11.33 ± 0.94	19.67 ± 0.83	22.33 ± 1.15	4.67 ± 0.58
E	NP	100	13.67 ± 0.83	22.67 ± 1.52	24.33 ± 0.58	6.33 ± 0.58
F	PBS	−	Undetected	Undetected	Undetected	Undetected

Note: BABL/c mice (female, 6–8 weeks old) randomly were divided to 6 groups. Mice were anesthetized, followed by immunized intranasally 3 times with 2 weeks interval containing different doses of NP or TAT-NP. The control group was received PBS. Blood samples of three mice in each group were collected on day 14, 28 and 42 to detected NP specific IgG. NAL of three mice were collected on day 42 to evaluate IgA. End-point ELISA titers were expressed as the reciprocal of the highest sample dilution that yielded an OD ≥ mean+2 × SD of control mouse serum. Results are expressed as mean ± SD of three tested mice in each group. The *t*-test was used to determine difference in antibody titers. ^a^ Displays statistically significant difference compared with the same dose NP group (*P* < 0.05).

IgA is the major antibody produced by a mucosal immune response, mainly secreted by B cells in the lamina propria of mucosa, while the influenza virus is a pathogen that infects via the respiratory tract. Therefore, IgA might play an important role in defense against influenza virus infection. The IgA in the NAL of mice after three vaccinations was detected. The results (Table 2) showed that a high titer of IgA antibody was produced in all TAT-NP and NP groups, and the titer of IgA induced in the same dose TAT-NP group was significantly higher than that in the NP group (*P* < 0.05). This result shows that TAT-NP could induce a better local mucosal immune response.

### TAT-NP effectively induced cellular immune responses in mice

Antigen-specific T lymphocytes play an important role in the generation and regulation of effective immune responses. Therefore, we prepared mouse spleen cell suspensions two weeks after the last vaccination. The number of IFN-γ-secreting splenic T lymphocytes was detected using ELIspot technique, with MHC-I and MHC-II epitope peptide pools from NP protein as the stimulants respectively. As shown in Figure 2(D), TAT-NP could significantly increase the number of antigen-specific IFN-γ-secreting CD8^+^ T cells (*P* < 0.05).

### TAT-NP provides protection against lethal doses of heterosubtypic influenza virus challenge in mice

**Figure 3.**
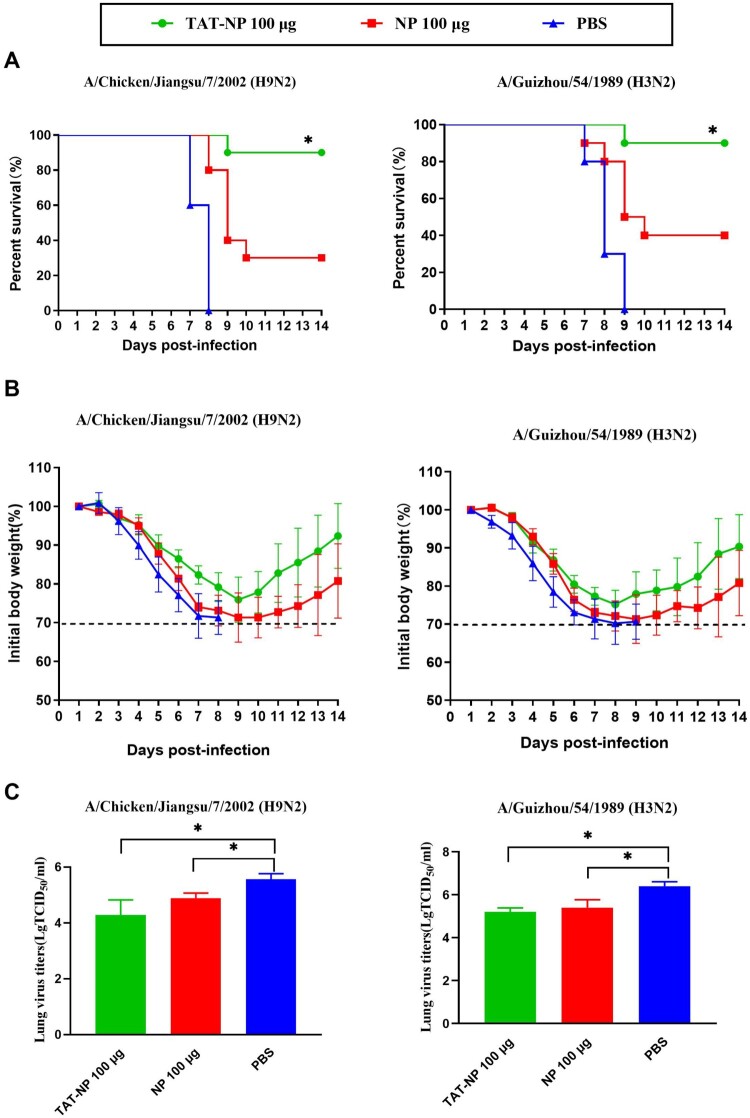
Protection of vaccinated mice against challenge with H9N2 and H3N2. BALB/c mice (*n* = 16 per group) were immunized intranasally on day 0, 14 and 21 with NP or TAT-NP, whereas control animals received PBS. All groups were challenge on day 42 with 10 × LD_50_ A/Chicken/Jiangsu/7/2002 (H9N2) or A/Guizhou/54/1989 (H3N2). The survival rates (A) and body weights changes (B) of the mice (*n *= 10 per group) were measured daily from the date of the challenge to 14 days after the challenge. The error bars represent the SDs. The survival rates were evaluated by log-rank (Mantel–Cox) test. * indicates significant differences compared with the same dose NP group (*P *< 0.05). The lung virus titers (C) are expressed as mean ± SD of three tested mice in each group. The *t*-test was used to determine difference in viral load. * indicates significant differences (*P *< 0.05).

We investigated the potential of TAT-NP as a candidate universal vaccine to provide cross protection. Mice were vaccinated with 100 μg of TAT-NP or NP three times at 2-week intervals and infected intranasally with 10 × LD_50_ A/Chicken/Jiangsu/7/2002 (H9N2) or 10 × LD_50_ A/Guizhou/54/1989 (H3N2) respectively 2 weeks after the last vaccination. Three mice from each group were examined for the virus titer in lungs three days after the challenge, and the remaining 10 mice were monitored for weight loss and survival rate. Mice infected with H9N2 or H3N2 in each group showed such clinical symptoms as pilomotor fur, quivering and weight loss in varying degrees. As shown in Figure 3, when challenged with H9N2, 90% (9/10) of mice vaccinated with TAT-NP were survived, while survival of NP group was only 30%. For the mice infected with H3N2, the survival of TAT-NP group was up to 90%, while survival of NP group was only 40%, indicating that TAT could enhance the protective immunity induced by NP.

As shown in Tables 3 and 4 and Figure 3, the results showed that both TAT-NP and NP could effectively inhibit virus replication. The above results indicate that the protein transduction domain TAT could increase the ability of NP to provide protection against lethal doses of heterosubtypic influenza virus challenge in mice, and that TAT-NP could be used as a candidate universal influenza vaccine.

**Table 3.** Protection of mice against lethal heterosubtypic H9N2 challenge by intranasal administration of TAT-NP or NP.

Group	Immunogen	Dose (μg)	Lung virus titers (Log_10_TCID_50_/mL)	Survival (survivors/total)	Challenge virus
A	TAT-NP	100	4.28 ± 0.54^b^	9/10^ab^	A/Chicken/Jiangsu/7/2002 (H9N2)
B	NP	100	4.89 ± 0.18^b^	3/10^b^	A/Chicken/Jiangsu/7/2002 (H9N2)
C	PBS	−	5.56 ± 0.20	0/10	A/Chicken/Jiangsu/7/2002 (H9N2)

Note: BABL/c mice were randomly divided into three groups. Mice were immunized intranasally with NP or TAT-NP vaccine, whereas control animals received PBS. Two weeks after the last immunization, mice were challenged with a lethal dose (10 × LD_50_) of influenza A/Chicken/Jiangsu/7/2002 (H9N2) virus. BALF from three mice in each group were collected 3 days post-infection for virus titration in the lungs respectively. The survival rate of mice (*n *= 10) was determined at 14 days post-infection. The *t*-test was used to determine difference in viral load. The survival rates were evaluated by log-rank (Mantel–Cox) test.

^a^Displays statistically significant difference compared with the same dose NP group (*P* < 0.05).

^b^Displays statistically significant difference compared with the PBS group (*P* < 0.05).

**Table 4.** Protection of mice against lethal heterosubtypic H3N2 challenge by intranasal administration of TAT-NP or NP.

Group	Immunogen	Dose (μg)	Lung virus titers (Log_10_TCID_50_/mL)	Survival (survivors/total)	Challenge virus
A	TAT-NP	100	5.20 ± 0.19^b^	9/10^ab^	A/Guizhou/54/1989 (H3N2)
B	NP	100	5.40 ± 0.37^b^	4/10^b^	A/Guizhou/54/1989 (H3N2)
C	PBS	−	6.40 ± 0.21	0/10	A/Guizhou/54/1989 (H3N2)

Note: BABL/c mice were randomly divided into three groups. Mice were immunized intranasally with NP or TAT-NP vaccine, whereas control animals received PBS. Two weeks after the last immunization, mice were challenged with a lethal dose (10 × LD_50_) of influenza A/Guizhou/54/1989 (H3N2) virus. BALF from three mice in each group were collected 3 days post-infection for virus titration in the lungs respectively. The survival rate of mice (*n *= 10) was determined at 14 days post-infection. The *t*-test was used to determine difference in viral load. The survival rates were evaluated by log-rank (Mantel–Cox) test.

^a^Displays statistically significant difference compared with the same dose NP group (*P* < 0.05).

^b^Displays statistically significant difference compared with the PBS group (*P* < 0.05).

## Discussion

Current influenza vaccination strategies typically elicit strain-specific immune responses with limited cross-reactivity to heterosubtypic viruses, and are therefore poorly equipped to protect against antigenically drifted viruses and newly emergent influenza viruses. Therefore, a universal influenza vaccine might play an important role in influenza prevention and control in the future. The high conservation of NP among influenza A viruses and the broad spectrum of immune responses generated among different influenza viruses by the CTL response have enabled NP to become an attractive candidate for universal influenza vaccine [23]. Our previous studies suggested that NP-based vaccines can provide partial protection, while single NP vaccines could achieve the desired protection only after multiple vaccinations [19,21]. In this study, we performed fusion expression of the protein transduction domain TAT and NP to enhance the transmembrane penetration into cells. The TAT-NP induced enhanced NP-specific CD8^+^ T cells and immune protection in mice. This vaccine not only provided better protection against homologous virus but also had good cross-protection effect with heterosubtypic viruses.

TAT is a short peptide derived from 47 to 57 amino acids (YGRKKRRQRRR) of HIV-1 transactivator protein, which has now been demonstrated to could mediate protein transmembrane effect [24]. Most protein transduction domains (PTD) are positively charged peptide. The PTD can interact with cell surface glycosaminoglycans (GAGs), which are negatively charged glycoproteins [25]. This PTD-cell surface GAGs electrostatic interaction induces internalization of exogenous antigens into the cytoplasm [26]. The PTD-antigens are internalized by antigen presenting cell (APC) with micropinocytosis-mediated, clathrin-mediated or caveolin-mediated endocytosis [27]. It is commonly recognized that the vaccine antigens as exogenous antigens are mainly recognized and presented by APC via the MHC-II pathway to activate the CD4^+^ T cells, while endogenous antigens can be presented via the MHC-I pathway to activate the CD8^+^ T cells. TAT-PTD can promote exogenous antigens to penetrate the cells and present to MHC-I molecules through cross-presentation [28,29]. Internalization antigens are degraded through proteasome/ubiquitin and transported to endoplasmic reticulum (ER) through transporter associated with antigen processing (TAP) for loading on MHC-I molecules [30]. We used TAT as a tool for the efficient delivery of antigen to APC and to induce more antigen specific CD8^+^ T cells. In present study, the result showed that TAT-NP elicited enhanced level of IFN-γ-secreting CD8^+^ T cells in mice. The data indicates that the protection provided by TAT-NP is mostly mediated by CD8^+^ T cell responses against the conserved NP antigen.

In this study, we successfully expressed TAT-NP proteins using a prokaryotic expression system and evaluated the immunogenicity and protection of TAT-NP against the influenza virus in mice. Serological results showed that the recombinant TAT-NP induced a high titer of NP-specific antibody in mice after three vaccinations. The results showed that the titer of NP-specific IgG antibody in mice increased with the frequency of vaccination. At the same time, the vaccine had good dose dependency among different groups, and the titer of IgG increased with the dose. In addition, the analysis of serum antibody subtypes showed that the IgG2a/IgG1 ratio in the TAT-NP group was significantly higher than that in the NP group, indicating that TAT-NP may induce a more pronounced Th1-biased immune response.

However, there has been controversy over the role of NP-specific antibodies in the body’s defense against an influenza virus infection. Although anti-NP IgG has no neutralizing activity, their importance should not be neglected. Previous study demonstrated that after the mice vaccinated with NP protein were infected with different subtypes of influenza viruses, the vaccine could speed up the clearance of virus in mice and reduce mouse mortality [19]. Adoptive immunization of naive mice with serum of mice vaccinated with NP protein could provide the mice with antibody-dependent protection, contributing to accelerated virus clearance in mice. However, this protection is significantly declined on the B cell deficient mice vaccinated with NP protein [31]. Unlike neutralizing antibodies, NP-specific antibodies cannot neutralize the virus and prevent the virus entry into host cells [32,33]. NP in various forms can be detected in the supernatant of MDCK cells infected with influenza virus and in the nasal wash of mice infected with influenza virus, including monomer, polymer and complex formed with RNA or polymerase, so that NP-specific antibodies have sufficient opportunity to interact with NP to exert antiviral immune responses [34]. In addition, investigators also found NP expression on the surface of influenza virus-infected cells [35]. NP-specific antibodies might eliminate virus-infected cells via the complement-dependent pathway by interacting with NP on the cell surface. It has also been shown that anti-NP IgG acts primarily through FcR (Fc receptors) and CD8^+^ T cells. NP-specific antibodies exert their antivirus effect mainly through FcR in μMT mice. FcR primarily acts through three different subtypes of IgG antibodies, with IgG2a playing the greatest role in virus clearance. In addition, the antibody interaction with FcR can also increase activation and antigen presentation of NK cells. Another important mechanism by which NP-specific antibodies exert their antivirus effect is the dependence on T cells, particularly CD8^+^ T cells [32,33,36]. This also explains why NP-specific antibodies cannot provide protection in mice deficient in both T and B cells.

IgA is the major antibody of the mucosal immune response, mainly synthesized by B cells in the lamina propria of mucosa. Mucosal IgA can also play a particularly important role since the influenza virus is a respiratory pathogen, colonizing trachea, bronchi and pulmonary alveoli as sites of viral replication. IgA secreted on the mucosal surface forms a protective layer that can neutralize pathogens and prevent invasion into the mucosa. In this study, we also evaluated the level of NP-specific IgA antibodies in the respiratory mucosa. The results showed good correlation between the survival rate of mice and the level of mucosal NP-specific IgA. It may exert the antivirus effect through intracellular neutralization. It has been shown that IgA can be internalized by the polymeric immunoglobulin receptor (pIgR) on the surface of epithelial cells, bind to the newly generated viral proteins in the cells, and inhibit virus assembly, thus resisting the virus infection [37,38]. A recent study showed that IgA antibody may mediate protection by intracellular neutralization in rotavirus [39]. Mucosal NP-specific IgA may recognize the envelope proteins as well as the internal proteins of the virus as is the case of influenza NP [40].

In conclusion, we described a candidate universal vaccine based on the conserved protein NP and protein transduction domain TAT. The recombinant vaccine induced potent humoral and cellular immune responses in mice. The TAT-NP could provide protection against lethal doses of homologous and heterologous influenza virus challenge. The mechanism of TAT enhancing the transmembrane ability and immune response of NP deserves further study. Taken together, this study demonstrated that TAT-NP is a promising to develop a universal influenza vaccine.

## Supplementary Material

Supplemental Material
