# Supplementary material for: Protein transduction domain-mediated influenza NP subunit vaccine generates a potent immune response and protection against influenza virus in mice
Source: Emerg Microbes Infect. 2020 Sep 2;9(1):1933–42. doi: 10.1080/22221751.2020.1812436 (PMC8284974; doi:10.1080/22221751.2020.1812436)
Supplement: Supplemental Material [file TEMI_A_1812436_SM6189.docx]

**Supplemental Material**

**
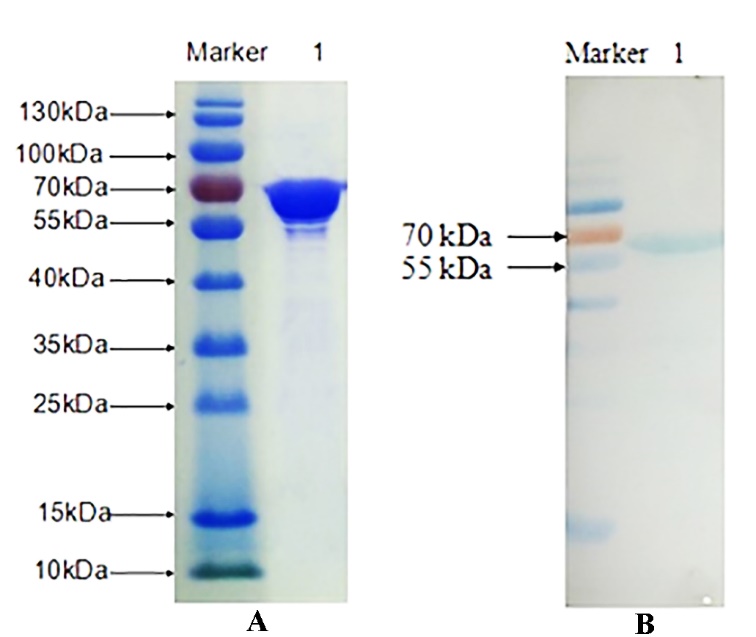
**

**Figure S1. SDS-PAGE and western-blot of recombinant TAT-NP.** Affinity-purified, sterile-filtered TAT-NP was fractionated by SDS-PAGE under reducing conditions and stained with Coomassie Blue (A). The electrophoresed proteins were transferred to a PVDF membrane on which TAT-NP was detected with an anti-His tag antibody (B).


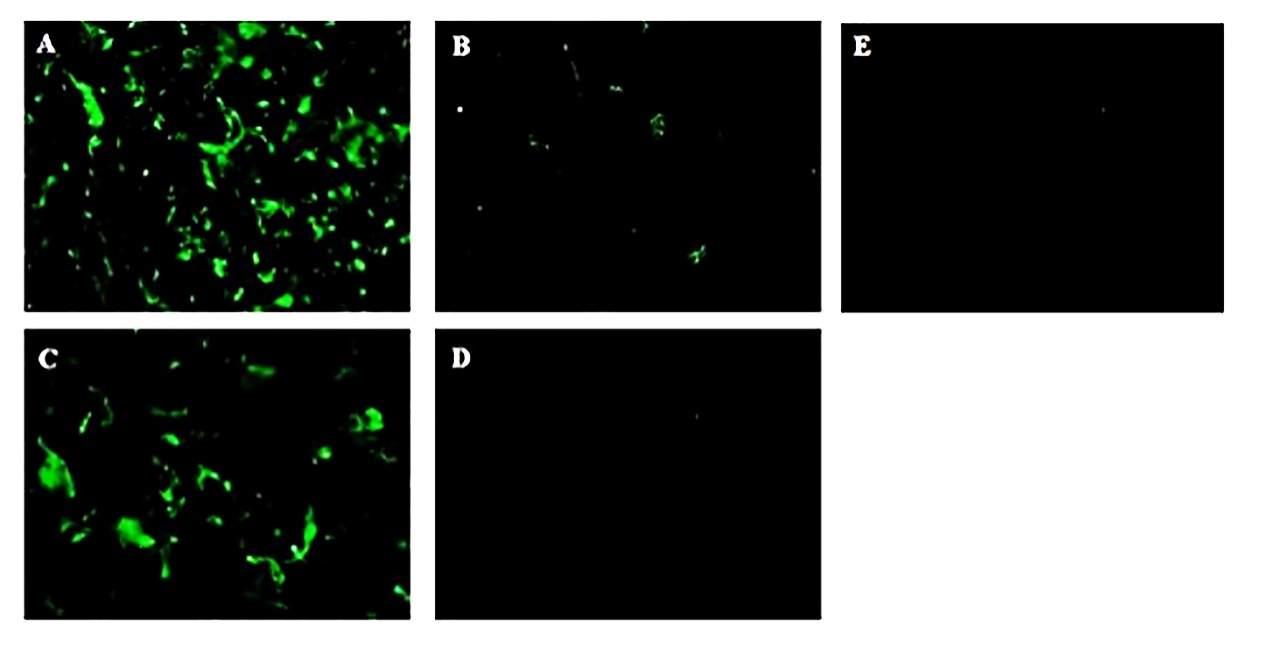


**Figure S2. Fluorescence microscope of 293T cell treated with TAT-NP or NP.** 293 T cells were incubated with either TAT-NP 20μg/mL (A), B. NP 20μg/mL (B), TAT-NP 10μg/mL (C), NP 10μg/mL (D), PBS(E) at 37℃ for 2h. The cells were then washed three times with PBS and fixed with 4% Paraformaldehyde for 30min.


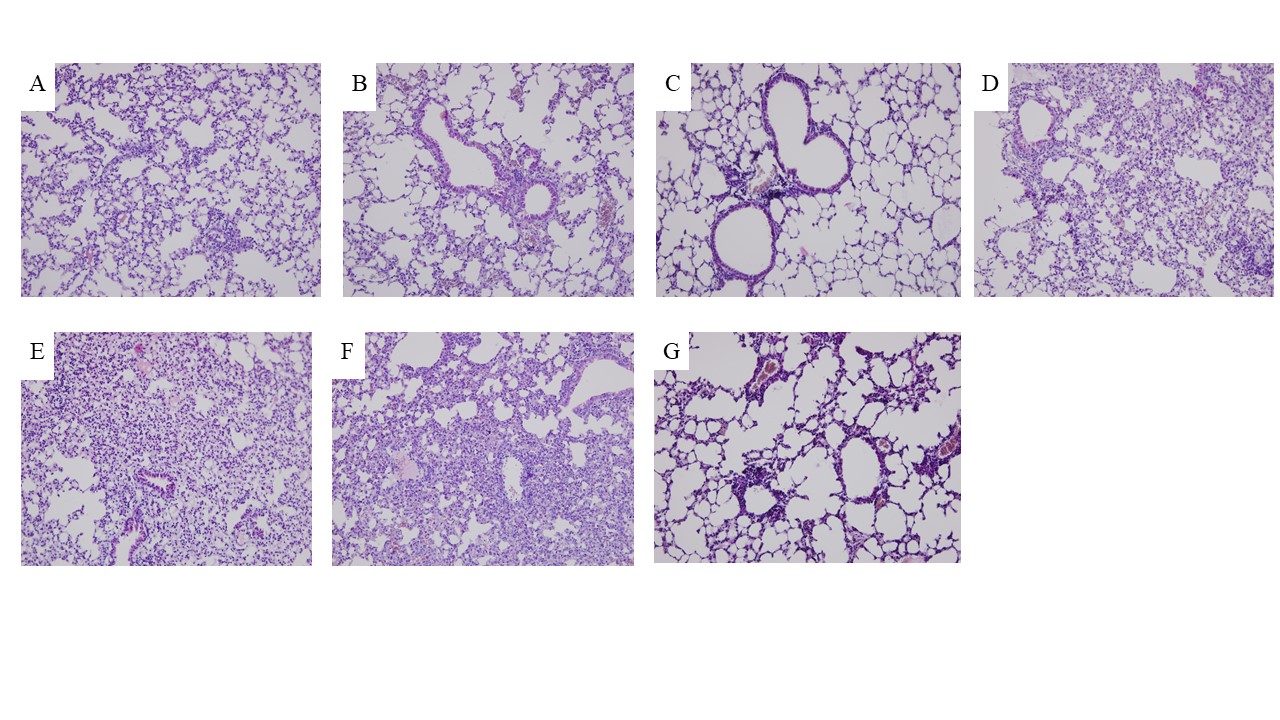


**Figure S3. Histological lesions of mice 3 days post-challenge.** Mice were intranasally administered with 10 μg TAT-NP (A), 30 μg TAT-NP (B), 100 μg TAT-NP(C), 30 μg NP(D), 100 μg NP(E) , and PBS (F) respectively, the Histopathological changes in lung of vaccinated mice following challenge with influenza A/PR/8 virus. the normal mice unchallenged (G) was as the normal control. Lung tissues were collected 3 days after challenge. Representative images of histopathological damage from H&E-stained lungs in three mice per group. Representative photographs are presented (original magnifications: 100×).
